# Embodied carbon quantification of luminaires using life cycle assessment and CIBSE TM65 methodologies: A comparison case study

**DOI:** 10.1111/jiec.13449

**Published:** 2023-10-13

**Authors:** Irene Mazzei, Ruth Saint, Alistair Kay, Francesco Pomponi

**Affiliations:** 1 School of Engineering and the Build Environment, Edinburgh Napier University, Merchiston Campus, 10 Colinton Road, Edinburgh, EH10 5DT United Kingdom of Great Britain and Northern Ireland; 2 Stoane Lighting Ltd., Loanhead, United Kingdom of Great Britain and Northern Ireland; 3 Cambridge Institute for Sustainability Leadership (CISL), University of Cambridge, The Entopia Building, 1 Regent St, Cambridge, CB2 1GG United Kingdom of Great Britain and Northern Ireland

**Keywords:** assessment methodologies, built environment, climate change, embodied carbon, industrial ecology, life cycle assessment

## Abstract

The European legislative landscape on sustainability is steadily growing to meet objectives to reach net zero targets by mid-century. In this context, companies and manufacturers may soon be legally required to provide quantification of the environmental impact of their products and services. A key challenge is applying a consistent and robust methodology that ensures comparability between assessments made by different companies, as there is still fragmentation among environmental impact reporting methodologies. The objective of this study is to analyze and compare the results of two different methodologies for the calculation of the embodied carbon in lighting products using the cradle-to-grave approach. One is the globally known life cycle assessment (LCA) method, and the other is Chartered Institution of Building Services Engineers Technical Memorandum 65’s (TM65) mid-level calculation methodology, which has been specifically tailored to building services. The two were applied to six different luminaires to evaluate their differences. Results show that the values of the embodied carbon calculated with TM65 are higher than those calculated with LCA and that the weight of the products plays a crucial role in creating discrepancies between the two methodologies.

**Supplementary Information:**

The online version of this article (doi:10.1111/jiec.13449) contains supplementary material, which is available to authorized users.

## INTRODUCTION

Greenhouse gas (GHG) emissions need to be urgently reduced to limit the impact of the global warming phenomenon and maintain the average temperature variation below the 1.5°C threshold (IPCC, 2019). To achieve this, regulations from the European Green Deal impose a reduction of GHG emissions, increase in renewable energy use, and an improvement in energy efficiency (European Commission, 2019). Despite some positive outcomes reached so far, the sixth IPCC report states that current efforts may not be enough to remain below the 1.5°C warming threshold (IPCC, 2023). Numerous initiatives are being put in place by governmental institutions to ensure that sustainability standards are met by companies and industries. An example of this is the adoption of the new directive proposal of the European Parliament and of the Council on substantiation and communication of explicit environmental claims which will require companies to provide quantification and assessment of their environmental claims (European Parliament, 2023). The aim of the initiative is to make claims reliable and verifiable across the EU, thus fighting the “greenwashing” phenomenon.

Lighting accounts for 15% of global electricity consumption and 5% of total global GHG emissions, as reported by the UN Environment Programme (UN Environment Program, 2017), thus minimizing its impact can be a contributing solution to tackle climate change. Although the energy in use accounts for the highest proportion of the total environmental impact of lighting products (Denneman & de Stoppelaar, 2015; Tähkämö et al., 2014; Wu & Su, 2021), improving the energy efficiency is not the only way to tackle the problem. A report by the Ellen MacArthur Foundation (EMF) (2021) highlighted that a great effort is being put into developing more energy-efficient strategies; however, it is also important to keep in mind the embodied emissions of products. The report shows that, for generic industrial products (i.e., cement, steel, food, plastic, and aluminum), an average portion equivalent to 45% of total GHG emissions is associated with manufacturing processes. Additionally, the relevance of embodied over operational emissions will also grow as light sources are expected to become progressively more energy efficient (International Energy Agency [IEA], 2023), and the carbon intensity of the electricity grid decreases (One Click LCA Ltd., 2021).

The life cycle assessment (LCA) methodology is used to quantify the environmental impact of products and services. The LCA must comply with standards ISO 14040:2006 (CEN, 2020a), ISO 14044:2006 (CEN, 2020b), and EN 15804 A2:2019 (CEN, 2019). To ensure consistency of the assessment for products belonging to the same category, product category rules (PCRs) should also be followed. LCA can quantify the environmental impact of each life cycle stage of a product system, from the material extraction to the end of life. LCA studies on luminaires have been carried out covering various aspects of these products, especially related to the energy-saving theme (Casamayor et al., 2018; Clarke-Sather et al., 2016; Elijošiutė et al., 2012; Ferreira et al., 2021; Principi & Fioretti, 2014; Tähkämö et al., 2012; Wang, Su et al., 2022; Wu & Su, 2021). However, to the best of our knowledge, there are no academic publications focusing on the quantification of the embodied carbon deriving from the production of luminaires.

Despite old (Weidema, 2014) and new criticism (Schaubroeck, 2022), LCA is globally recognized as a robust methodology and it can be applied to evaluate the impact of design decisions or changes applied to the manufacturing process of a product, thanks to the high granularity and flexibility of the methodology. However, the LCA calculation methodology is still complex and requires expert knowledge to carry out properly (Wang, Tao et al., 2022). Additionally, although PCRs exist to limit the variability of the model and ensure more consistency in the results, users’ choices still play a major part in the analysis (Scrucca et al., 2020). Moreover, due to the complexity and costs associated with the publication of the results in the form of Environmental Product Declarations (EPDs), there is still a large number of manufacturers that are not engaged with environmental impact quantification. Regarding lighting products, the number of EPDs available represents only a small portion of products in the industry (PEP Ecopassport, n.d.).

Technical Memorandum 65 (TM65) (Hamot & Bagenal George, 2021) is an embodied carbon calculation methodology released in 2021 by the Chartered Institution of Building Services Engineers (CIBSE), aimed at mechanical, electrical, and public health (MEP) products and systems. According to the definition provided by the Royal Institution of Chartered Engineers (RICS), the embodied carbon of a product is defined as the GHG emissions (over 100 years) associated with material extraction, transport, manufacturing, distribution, and disposal of the product, according to the cradle-to-grave system boundaries (RICS, 2017). The authors clarify that the TM65 methodology is not intended to replace the use of EPDs, as these should be the preferred form of environmental communication. With this purpose, TM65 also provides guidance on how to produce and use EPDs. However, to bridge the gap between industry and environmental impact quantification and facilitate the understanding of the whole-life carbon of MEP systems, TM65 was introduced as a simpler methodology for the industry to become familiar with the concepts of embodied carbon quantification, until manufacturers are able to consistently provide EPDs for their products.

In Appendix D.3.3 of TM65, comparisons between embodied carbon values obtained from EPDs and the basic calculation level of TM65 are reported (Hamot & Bagenal George, 2021). The values of embodied carbon estimated with TM65 are higher than the ones reported from EPDs for most product types, due to the conservative nature of the estimate provided by TM65. The only two lighting products reported (exit lights) fall in this category as well, with a TM65 embodied carbon, respectively, 88% and 26% higher than the corresponding EPD values. To the best of our knowledge, this is the only reported direct comparison of embodied carbon values between TM65 and LCA results for lighting products. The aim of this study is for the first time to compare harmonized assessments of luminaire products done with TM65 against an LCA with equivalent scope. For completeness, a full LCA, compliant with EN 15804 A2:2019, has been carried out to enrich the findings. The results will show the extent to which TM65 can at least approximate findings produced with LCA. With this, the building services community in general, and its luminaire sub-community in particular, will be better equipped to critically appraise the validity of the results of TM65 assessment. This will also help understand which life cycle stages contribute the most in creating differences between results obtained using the two methodologies. Further, the findings reported in this study could be used as a starting point for a revision of TM65 with the aim of providing more accurate results. In addition to this, lighting manufacturers choosing to use TM65 for the embodied carbon quantification of their products will have a clearer idea of how close the values are to the results of an LCA analysis. Ultimately, shedding more light on the alignment between industry-led assessment schemes and more rigorous scientific analyses will help improve the relevance and robustness of the former and drive more meaningful climate action. The authors also believe that this study, conducted on six product systems made by the same manufacturer using the same production process, may lead the way to wider analyses, which may include comparisons between different manufacturers, designs, and production strategies.

## METHODS

The LCA and TM65 analyses in this study have been carried out on six product systems: three standard products chosen from the company’s catalog (Stoane Lighting, 2023) and three bespoke chandeliers manufactured by Stoane Lighting.

### TM65 methodology

In the absence of an EPD, where TM65 needs to be applied, the methodology requires information that can be collected from the manufacturer of a product. It is possible to apply two different calculation levels to the data: a basic level calculation, in the case of low-detail data provided by the manufacturer or a mid-level calculation where information is provided with a higher level of detail, including the estimated portion of energy associated with the manufacture of the product. For this study, the mid-level method was applied.

For an in-depth explanation of the methodology, the reader should refer to the TM65 guideline document (Hamot & Bagenal George, 2021).

### Product systems

Details of the products are reported in Table 1. The products were selected based on their material composition, manufacturing processes, and size, to ensure the inclusion of a variety of characteristics in the analysis.

**TABLE 1:** Technical properties of the standard products and bespoke projects.

		Standard			Bespoke		
Quantity	Unit	Fitting 1	Fitting 2	Fitting 3	Chandelier 1	Chandelier 2	Chandelier 3
LED type		Module			Strip and modules	Strips	Modules
Luminaire power	W	1.8	10.8	15.2	2333.0	1761.5	3034.2
Lifetime	h	100000			100000		
Luminaire weight	kg	0.09	0.44	1.19	306.36	435.55	670.34
Size (*W* × *D* × *H*)	m	0.05 × 0.03 × 0.04	0.2 × 0.06 × 0.1	⌀0.3 × 0.2	⌀11.6 × 2.2	5.4 × 2.4 × 4.2	14.5 ×8.9 × 7.8

**FIGURE 1:**
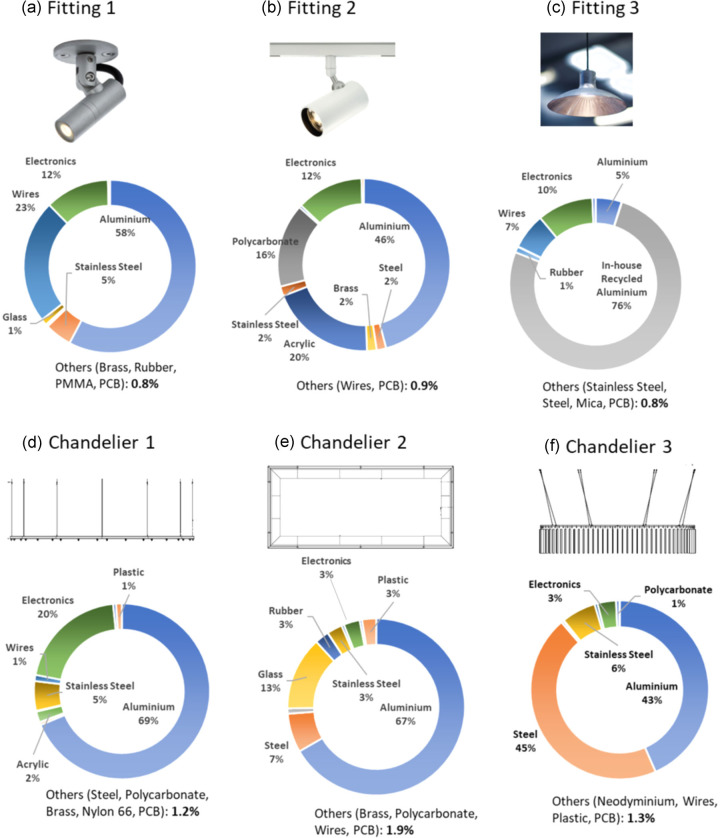
Catalog products (a, b, c) and bespoke chandeliers (d, e, f) and their material composition.

The standard products are shown in Figure 1. Fitting 1 and Fitting 2 were produced starting from aluminum bars and extrusions (with minimum 70% recycled content) which underwent manufacturing operations to fabricate each part. Anodizing was carried out on the components, and, in the case of Fitting 2, the product was also sprayed using a powder-based coat. After this process the product was dried in a gas oven. Fitting 3 was produced starting from aluminum scraps derived from in-house collected manufacturing waste. The scraps were placed in a crucible and melted in-house in a gas forge. The aluminum was then poured into a mold via gravity die casting and the resulting pendant was further processed to obtain the final product. The assembly of the products was carried out in-house using manual tools.

Chandelier 1 was manufactured from aluminum as the primary material. The chandelier was shipped to the customer site overseas by plane. Chandelier 2 was mostly made of aluminum and glass, with most manufacturing operations carried out in-house. Sections of the chandelier were anodized and painted. Chandelier 3 was mostly made of steel and aluminum; laser cutting was intensively used during manufacturing. Chandelier 1 and Chandelier 3 also required rolling of metal bars for their structure.

### Goal and scope definition

The goal of the study is to calculate the embodied carbon of the products with TM65 and LCA methodologies, with the aim of comparing the results. This will allow an understanding of which factors are causing differences in the values of embodied carbon obtained with the two methodologies.

The declared unit was defined independently for each product, as a luminaire providing a luminous flux of X lumens during a reference life of 25 years. This choice was made considering the goal of the study of comparing assessment results obtained from two different methodologies, and not across different products. Therefore, the definition of a functional unit would not bring any additional value to the comparison and was not applied. The values of X in the declared unit definition for the products are reported in Table S1.1 in Supporting Information S1.

### System boundaries

The LCA was carried out following rules dictated by standards ISO 14040:2006 (CEN, 2020a), ISO 14044:2006 (CEN, 2020b), and EN 15804 (CEN, 2019) and the TM65 analysis was carried out according to its mid-level calculation method (Hamot & Bagenal George, 2021). The cradle-to-grave approach was used for both calculation methodologies and the life cycle stages considered in the analysis included:

- The raw material extraction, transport to factory, product manufacturing, and transport to site (stages A1 to A4).
- One round of replacement of light sources and power supplies (stage B3).
- The transport to waste facility, waste treatment, and disposal (stages C2 to C4).

Despite some of the products being sold outside the United Kingdom, the end-of-life (EoL) scenario modeled for this study was based on statistics on recycling, landfill, and incineration rates of relevant materials in the United Kingdom with the aim of predicting a “best case scenario.” The scenario selected shows higher recycling rates compared to the European target and the value of TM65. This choice was made considering that manufacture is carried out in the United Kingdom and most products are sold in the United Kingdom; additionally, for the aim of this study, the application of a single EoL scenario was selected to simplify the analysis and focus on comparing methodologies. For a more accurate assessment, the authors suggest referring to different EoL scenarios for products sold outside the United Kingdom, for instance considering information of eco-organizations and minimum recovery targets specified in the Directive 2012/19/EU (European Parliament, 2012).

The system boundaries excluded:

- The construction/installation stage (A5).
- The use (B1), maintenance (B2), replacement (B4) stages.
- The deconstruction/demolition stage (C1).

These choices were made considering that the excluded life cycle stages are not applicable or provide negligible impact in luminaires. Additionally, the embodied carbon definition excludes the operational energy (B6) and any evaluation of benefits and loads beyond the life cycle (stage D), according to the cradle-to-grave approach. The life cycle stages of a product system as described in EN 15804 are shown in Figure S1.1 in Supporting Information S1.

Some exclusions from the system boundaries described in EN 15804 were made with the aim of aligning the scope of the LCA with TM65. These included packaging, manufacturing waste, and installation (A5) and deconstruction (C1) stages. The corresponding flows are listed in Table S2.7 and Table S2.8 in Supporting Information S2, subsequently included in the LCA assessment to compare results obtained with a modified scope to a more comprehensive scope. The results of the LCA compliant with EN 15804 A2:2019 are reported in Supporting Information S2 (Table S2.9 and Table S2.10). The difference between global warming potential (GWP) (100 years) values from an EN 15804-compliant LCA and the LCA with modified scope applied in our study was on average 2.1% and always below 5% for all products (as shown in Table S1.11 in Supporting Information S1), therefore the LCA with modified scope was used for the comparison with TM65 results.

Information regarding stage B6 can be found in Section S.2 of Supporting Information S1. The lifetime of all product systems was considered as 25 years, with 4000 h of use per year, corresponding to a total of approximately 100,000 h.

**FIGURE 2:**
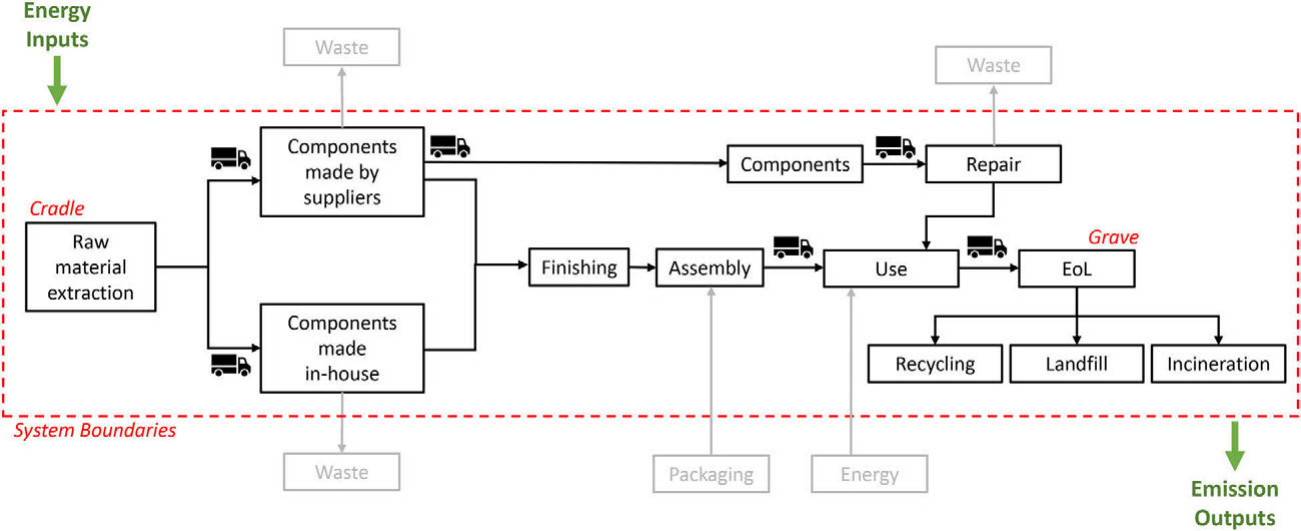
Flow diagram showing system boundaries of the product systems analyzed with life cycle assessment and Technical Memorandum 65.

The luminaires were evaluated including the light sources, drivers, power supplies, and cables in TM65 and LCA. The power supplies for the catalog products were included considering a fraction of their total mass, corresponding to the capacity necessary to power one fitting of each type (1/12 for Fitting 1, 1/2 for Fitting 2, and 1 for Fitting 3). A diagram showing the system boundaries and the included and excluded flows is represented in Figure 2.

### Life cycle inventory: Materials and processes

The analyses were carried out starting from the bill of materials for the catalog products and bespoke projects. The life cycle inventory (LCI) data related to stages A1 and A2 are collected in Table S2.1 for the bespoke projects and in Table S2.2 of Supporting Information S2 for the catalog products. Data on the manufacturing processes necessary for the LCA analysis of stage A3 were collected directly from the manufacturer (Stoane Lighting Ltd.), measuring the energy usage of the machines involved in the manufacturing processes, or they were estimated based on more generic data (e.g., provided by suppliers or sub-contractors). The LCI data for processes are shown in Table S2.3 for bespoke projects and in Table S2.4 for catalog products in Supporting Information S2. The following subsections provide a breakdown of the life cycle stages across the two methodologies.

#### Materials and transport in TM65 (A1–A2)

Material flows in TM65 are taken into account considering the masses of the products’ components and assigning the corresponding embodied carbon coefficient to each material. The coefficients used in the TM65 analysis are reported in Table S1.2 in Supporting Information S1. The material breakdown of products corresponded to at least 95% of the total product weight, as required by the TM65 methodology. To evaluate the emissions associated with the transport of the materials to the factory, a distance of 3000 km was considered, corresponding to a TM65 complexity category equal to 2, with emissions of 0.000132 kg CO_2_ eq./kg km (value used for all heavy goods vehicles) (UK Government, 2020).

#### Materials and transport in LCA (A1–A2)

Material flows in LCA are obtained considering the masses of the product’s components and excluding the mass of material waste produced during manufacturing operations, in order to align the methodology to TM65. In addition to this, it is possible to specify the processes applied to the raw materials or parts before reaching the final manufacturing site, which allows greater accuracy in the results. The transport of the raw materials and parts to the factory site was included in the LCA, representing stage A2 of the life cycle of the product. The transport distances considered in the model are the result of the sum of all materials/components provided by various suppliers.

#### Manufacturing in TM65 (A3)

The manufacturing stage (A3) in TM65 is calculated considering the energy and gas usage associated with making the product, based on its weight, in relation to the total production (8900 kg) and energy usage (35289 kWh for electricity and 50682 kWh for gas) of the factory. The embodied carbon coefficients for the production and distribution of electricity and gas used for manufacturing operations were used as provided by TM65 and are reported in Table S1.3 and the values of electricity and gas usage for every product estimated from the total usage and the total factory production are reported in Table S1.4 in Supporting Information S1. According to TM65 category assignment, luminaires belong to complexity category 2, which includes two rounds of manufacturing for these products.

#### Manufacturing in LCA (A3)

Data collection for manufacturing operations was carried out directly for the manufacturer, by analyzing each operation needed to manufacture the components in standard products and bespoke chandeliers. The current and voltage of the machines involved in the production processes were measured during typical operations and the values were used to calculate the apparent power of the machines. The time needed for the operations involved in the manufacturing of the products of interest was estimated based on data provided by the manufacturer. Where relevant, finishing procedures such as anodizing and painting were taken into account by estimating the mass of water, paint, electricity, and gas (propane) involved in the process for each product. The energy used for product manufacturing was considered as electricity from 100% renewable sources (based on electricity provider’s data) and evaluated based on statistics for Scotland in Q4 of 2020 (The Scottish Energy Statistics Hub, 2021) as the manufacturing facilities were entirely based in Scotland. The information on electricity production was used in compliance with EN 15804 A2:2019, which provides the possibility to include assumptions about electricity production (CEN, 2019). Gas involved in manufacturing operations was modeled as propane gas.

#### Transport to site in TM65 (A4)

To evaluate the carbon emissions associated with the transport of the products to the customer site, TM65 divides the product into four categories based on the manufacturing location: local, national, European, or global. For the purpose of this study, the products were considered as nationally manufactured when the installation site was in the United Kingdom (with a distance of 300 km by road), European in the case of Chandelier 2 installed in France (with a distance of 1500 km by road), and globally manufactured with a bespoke distance (5530 km by sea) in the case of Chandelier 1, as it was installed outside Europe.

#### Transport to site in LCA (A4)

The distribution of the standard products was evaluated considering London, United Kingdom as the customer site, based on marketing data provided by Stoane Lighting, showing that approximately 70% of the customers are based in London. For bespoke chandeliers, the travel distance was considered as the distance between the factory site (Edinburgh, Scotland) and the final customer (sites in United States, France, and England). Details regarding distribution data are reported in Table S1.5 in Supporting Information S1.

#### Repair (B3)

During the lifetime of the product (25 years or 100,000 h with 4000 h/year) the repair stage is accounted for considering one round of replacement of light sources and power supplies for each product. This choice was made considering 50,000 h as the typical lifetime of these components, which would limit the lifetime of a product and, therefore, make them likely to be changed. In TM65 the carbon emissions of the repair stage are evaluated considering the proportion of the product equivalent to the replaced components, multiplied by the emissions of material extraction, transport, and waste disposal stages. A similar approach was adopted in the LCA, considering the material flows of the components involved in the repair stage and the distances traveled to the manufacturing and the customer sites.

#### End-of-life in TM65 (C2–C4)

The EoL processes are described in TM65 as stages C2 to C4. Stage C2 (transport to waste processing facility) was evaluated as 100 km by truck. The emissions associated with the disassembly of the product in the waste processing stage, C3, were calculated considering the same energy used during the assembly stage in the factory with only one round of manufacturing. The recycling rates for different types of products needed for the calculation of emissions in stage C4 (waste disposal) are provided by TM65; for luminaires, 45% of the waste is recycled at the EoL and 55% is disposed in landfill. The CO_2_ emissions of landfill are considered as 0.0089 kg CO_2_ eq./kg waste (UK Government, 2020).

#### End-of-life in LCA (C2–C4)

The impact of the EoL stage was evaluated considering the same stages included in TM65. For stage C2, 100 km by municipal waste lorry (Municipal waste collection service by 21 metric ton lorry {CH}| processing | Cut-off, U in Ecoinvent) was used. The recycling rates of materials in luminaires were obtained from data published by Recolight (n.d.) and other sources (European Parliament, 2020; Jin et al., 2018) and they are reported in Table S1.6. Ecoinvent processes for the treatment of each waste stream are reported in Table S1.7 in Supporting Information S1.

### Life cycle impact assessment

The source for the LCI data was the Ecoinvent 3.7.1 database (Wernet et al., 2016). The environmental impacts were calculated using the SimaPro software, version 9.2.0.2 (*SimaPro*, n.d.) by PRe’ Sustainability (PRé Sustainability B.V., n.d.). The method used to assess the LCI is the CML-IA baseline method version 3.06 (CML-IA Characterisation Factors, 2016), which includes 11 impact categories, listed in Table S1.8 in Supporting Information S1. The embodied carbon values were obtained from the LCA results, considering only the GWP (100y) values, without stage B6. The results of the full LCA analysis are reported in section S.3 (Figure S1.2) of Supporting Information S1 and in Table S2.5 and Table S2.6 of Supporting Information S2.

## RESULTS AND DISCUSSION

### TM65 embodied carbon results

#### Standard products

**FIGURE 3:**
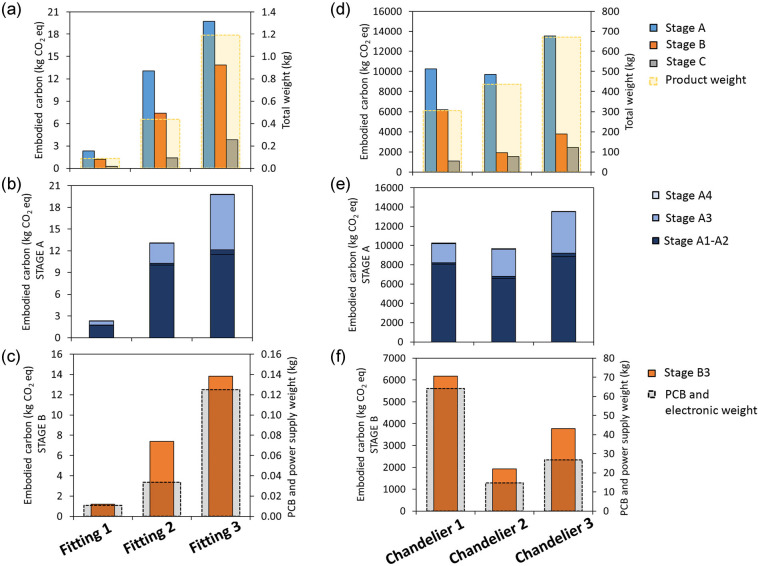
Embodied carbon for standard products (a, b, c) and bespoke chandeliers (d, e, f) calculated with Technical Memorandum 65 mid-level method. Total embodied carbon (a, d), values for stage A (A1-A4) (b, e); values for stage B (B3) (c, f). Underlying data for this figure are available in Table 2.

The results of the embodied carbon calculation carried out with the TM65 methodology for standard products are shown in Figure 3. In Figure 3a, the embodied carbon values are plotted with the total weight of the products, and a correlation can immediately be seen between the two: the heavier the product, the higher the embodied carbon values. This is due to the strong dependence of TM65 calculations on materials and components’ weights. Figure 3b shows that this is mostly dependent on the material extraction stage (A1); however, as the mass of the product increases, the manufacturing stage (A3) becomes more relevant, as a heavier product will correspond to higher energy/gas usage during manufacturing, according to the TM65 methodology. For Fitting 3, most of the aluminum comes from in-house produced aluminum scraps, therefore the proportion of impact associated with stage A1 will be lower, although the product’s mass is the largest of the three standard fittings. This is due to the use of secondary aluminum, from the company’s own manufacturing operations, bearing less environmental burdens compared to virgin aluminum. The transport stage (A4) provides only little contribution, as its impact is three orders of magnitude lower than the manufacturing stage. Stage B accounts only for the repair stage (B3) from an embodied perspective, considered as one round of replacement of light sources and electronic components during the lifetime of the product (25 years). Figure 3c shows the trend followed by stage B3, relative to the mass of the light sources and electronic components in the products, and as the masses of the latter increase, so do the embodied carbon values. Furthermore, the results show that stage A contributes the highest embodied carbon values to the total for each product, whilst stage C has the lowest impact.

#### Bespoke projects

The results of the TM65 embodied carbon calculations carried out on bespoke projects are shown in Figure 3. The total embodied carbon plotted relative to the total mass of the projects in Figure 3d shows a higher embodied carbon for Chandelier 1 compared to Chandelier 2, despite it being of a lesser mass. This is due to the material composition of the projects: in Chandelier 1, electronic components and light sources add up to over 20% of the total mass. These components have embodied carbon coefficients of 49 and 154 kg CO_2_ eq./kg, respectively, therefore they provide major contributions to the total embodied carbon values. This effect can further be noticed in Figure 3e,f, representing the embodied carbon of stages A and B. For stage B, the dependence on the electronic component and light source weights is evident: the high embodied carbon value of B3 for Chandelier 1 is due to the large mass of these components.

### LCA embodied carbon results

#### Standard products

**FIGURE 4:**
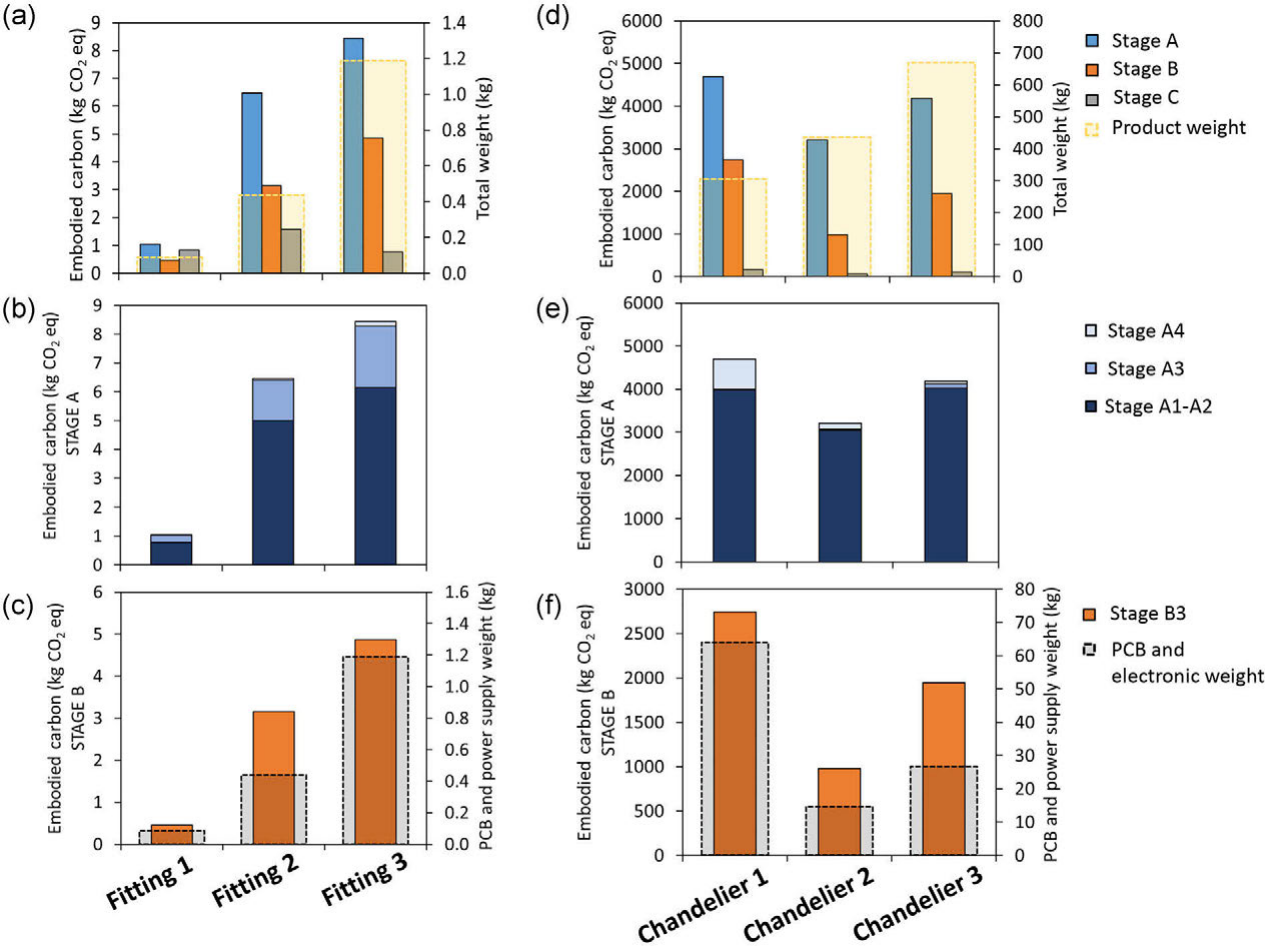
Embodied carbon for standard products (a, b, c) and bespoke chandeliers (d, e, f) calculated using life cycle assessment. Total embodied carbon (a, d), values for stage A (A1-A4) (b, e); values for stage B (B3) (c, f). Underlying data for this figure are available in Table 2.

The results of the embodied carbon calculated with LCA of standard products are shown in Figure 4. The trend observed for the results obtained in TM65 is also present in Figure 4a for the results obtained with LCA. Focusing on stage A1, Figure 4b reveals that the difference between Fitting 2 and Fitting 3 is minor, despite Fitting 3 being the heaviest. This can be explained considering that the aluminum used in Fitting 2 contains a 70% proportion of recycled aluminum, whilst in Fitting 3 98% of the aluminum used derives from in-house produced scraps. This contributes to lower the impact of the material extraction stage for Fitting 3. The impact calculated for stage B (Figure 4c) follows the trend of increasing embodied carbon with increasing light source and power supply weights.

#### Bespoke projects

The embodied carbon values calculated with LCA for bespoke chandeliers are shown in Figure 4. As with the observations in the TM65 analysis, the presence of a greater amount of electronic components and light sources contributes to the higher value for stages A and B (Figure 4d,f). However, a significant factor for Chandelier 1 is the transport-to-site stage (A4), as this chandelier was shipped from Scotland to the United States by plane. The carbon emissions of stage A4 alone correspond to 15% of stage A, as shown in Figure 4e. The impact of the manufacturing stage (A3) is negligible for bespoke chandeliers, as its value is considerably lower compared to stage A1. In the specific case of this chandelier, had it been installed in Scotland, its embodied carbon for the A stage would have been 15% lower, with no merit for anyone other than the luck of being “needed” close to its manufacturing location. This is important in LCAs within the built environment, as transportation impacts are often claimed to be extremely low and even negligible—which this specific example instead disproves.

### Direct comparison

**FIGURE 5:**
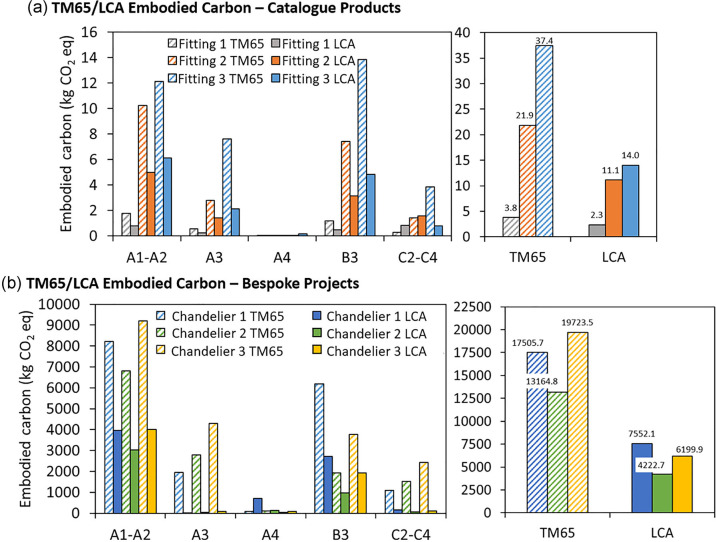
Embodied carbon values calculated with life cycle assessment and Technical Memorandum 65 mid-level methodologies, compared for each life cycle stage and as total values in standard products (a) and bespoke chandeliers (b). Underlying data for this figure are available in Table 2.

The values from the embodied carbon calculations for each life cycle stage, obtained with TM65 and LCA methodologies, are shown in Table 2 and plotted in Figure 5a,b.

**TABLE 2:** Values of embodied carbon for standard products and bespoke chandeliers calculated with the life cycle assessment and Technical Memorandum 65 mid-level calculation methodologies. Values are reported as the total and broken down by life cycle stage.

Standard products								
	Method	Unit	Total	A1–A2	A3	A4	B3	C2–C4
Fitting 1	TM65	kg CO_2_ eq.	3.8	1.8	0.6	0.6 × 10^−3^	1.2	0.3
	LCA		2.3	0.8	0.3	0.1 × 10^−1^	0.5	0.8
Fitting 2	TM65	kg CO_2_ eq.	21.9	10.3	2.8	2.9 × 10^−3^	7.4	1.4
	LCA		11.1	5.0	1.4	0.6 × 10^−1^	3.1	1.6
Fitting 3	TM65	kg CO_2_ eq.	37.4	12.1	7.6	7.8 × 10^−3^	13.8	3.8
	LCA		14.0	6.1	2.1	0.2	4.8	0.8

Bespoke chandeliers								
	Method	Unit	Total	A1–A2	A3	A4	B3	C2–C4
Chandelier 1	TM65	kg CO_2_ eq.	17505.7	8223.1	1959.2	41.9	6181.2	1100.3
	LCA		7552.1	3960.9	13.7	696.8	2725.9	154.8
Chandelier 2	TM65	kg CO_2_ eq.	13164.8	6807.5	2783.9	112.1	1932.4	1529.0
	LCA		4222.7	3025.4	28.5	159.8	975.5	120.4
Chandelier 3	TM65	kg CO_2_ eq.	19723.5	9205.3	4286.9	34.5	3773.4	2423.4
	LCA		6309.2	4027.3	92.1	119.5	1932.9	137.4

Figure 5a shows the results for standard products for each life cycle stage. For stages A1–A2, the embodied carbon values of the three fittings calculated with TM65 correspond to twice the values from the LCA. The same trend can be observed for the manufacturing stage (A3) for Fitting 1 and Fitting 2, whilst for Fitting 3 TM65 produces an embodied carbon value more than three times higher than the LCA value. This is explained considering that the manufacturing stage for the latter only consists of the aluminum smelting and casting, with only a few other manufacturing steps (such as sanding and polishing). However, Fitting 3 has the largest mass among the standard products, which as a consequence produces the highest embodied carbon value in TM65. These results demonstrate that the quantification of A3 based solely on the mass of the products may be misleading. Manufacturing processes may vary substantially between products, even for products belonging to the same category. The current TM65 approach is optimized to be adaptable to different product categories, however this causes it to be unsuitable to capture differences in manufacturing operations, often causing overestimation of the results. The same trend is observed for the repair stage (B3). The transport-to-site stage (A4) has a minor contribution in TM65, whilst the LCA analysis returns values two orders of magnitude higher. This is due to a more accurate estimate of the distances traveled by the products and their impact. The higher values obtained for the EoL stages (C2–C4) can be attributed to the presence of the incineration treatment in the EoL scenarios, which is not accounted for in TM65. The inconsistency between TM65 and LCA results for Fitting 3 is once more a result of the greater mass of the product. Overall, an overestimate of 47% and 65%, respectively, was found in TM65 compared to the LCA for Fitting 1 and Fitting 2 and of 91% for Fitting 3.

In bespoke projects, the discrepancies between the two methodologies are more evident, as shown in Figure 5b. Stages A1–A2 for the three chandeliers differ by over 100%, with TM65 showing the highest values of embodied carbon. A major difference is observed for stage A3 for all bespoke chandeliers, for which results calculated with LCA are 2 orders of magnitude lower than results obtained with TM65. The reason for this is related to (i) the large mass of the products and (ii) the method used in TM65 that correlates the mass to the energy and gas used during manufacturing. Additionally, some of the features of the manufacturing operations involved in the production of the chandeliers could not be accounted for in the LCA, such as the impact of the waste produced during metal rolling operations for Chandeliers 1 and 3. This technique generally produces considerable amounts of scraps, which would normally be included in an LCA and contribute to the environmental impact of the product. However, for this study, the need to consider system boundaries as consistently as possible for the two methodologies required manufacturing waste to be excluded from the system boundaries. Results of LCA carried out considering manufacturing waste flows are reported in Section S.4 of Supporting Information S1 (Figure S1.3), and of Table S2.9 and Table S2.10 in Supporting Information S2. The results of the weight dependence in TM65 can also be observed for stages B3 and C2–C4. On the other hand, the transport-to-site stage (A4) shows a reversed trend compared to the other life cycle stages, as the values calculated with LCA are higher than the embodied carbon values from TM65, especially for Chandelier 1, due to a more accurate representation of the impacts associated with this life cycle stage.

### Uncertainty analysis

**FIGURE 6:**
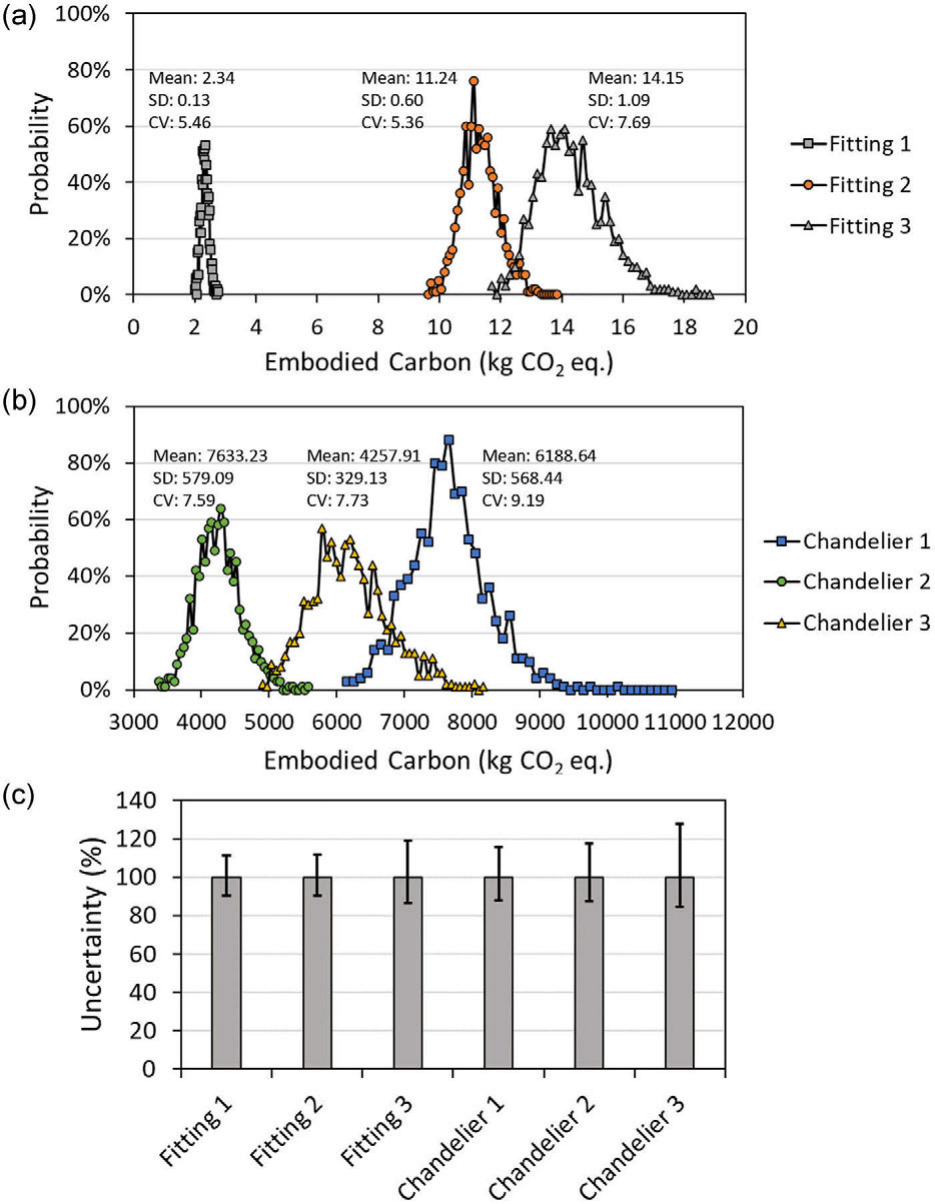
Results of Monte Carlo simulations carried out on catalogue fittings (a) and bespoke chandeliers (b); uncertainty on the embodied carbon results for each product (c). Underlying data for this figure are available in Table S2.11 in Supporting Information S2.

Uncertainties generated by data quality, methodological choices, and the impact assessment model were evaluated through Monte Carlo simulations (Barahmand et al., 2022). For each fitting and chandelier, the Monte Carlo simulation was executed 1000 times; the results of the analysis are reported in Figure 6. Figure 6a,b shows the distribution of embodied carbon results for the six products and the mean (in kg CO_2_ eq.), standard deviation (SD, in kg CO_2_ eq.), and coefficient of variation (CV, in %). The CV is calculated as the SD divided by the mean, and represents the variability across the values, in relation to the mean. From the results, it appears that bespoke chandeliers have on average higher CV than standard fittings. This aligns with expectations due to the higher variability of materials coming from more fragmented supply chains that are used in bespoke products. Overall, all CV values are below 10%, which generally indicates an acceptable level of dispersion. Figure 6c shows the uncertainty (%) on the embodied carbon results; the error bars represent the 95% confidence interval. The data used to generate the figures are reported in Table S2.11 in Supporting Information S2.

### Limitations and future research

The topic of embodied carbon calculation and reporting in the built environment has considerably gained relevance in recent years (Pomponi et al., 2018). This study contributes to the knowledge in the field of environmental impact quantification in the lighting industry by providing a direct comparison between the embodied carbon results using LCA and the industry-led methodology TM65, applied to a range of lighting products. This analysis has not been carried out before, and no studies have been found that focus on embodied carbon calculations for this category of products.

However, despite its novelty, some limitations are related to the use of process data and to the focus on GHG emissions and embodied carbon alone.

Regarding process data, this introduces uncertainty in the study as they can be highly incomplete for certain types of materials. On average, it was shown that process data for materials is 55% incomplete, but considerable inconsistency in system boundary coverage means that this incompleteness varies from 2% to 99% across materials and environmental flows (Crawford et al., 2022). The uncertainty analysis carried out through Monte Carlo simulations in this study and reported in Section 3.4 does not cover uncertainty related to the use of process data.

Additionally, embodied carbon (and GHG emissions in general) is one of the numerous environmental flows associated with the production of luminaires. This study does not focus on the other LCA impact categories, however the complete LCA results are reported in sections S.3 and S.4 of Supporting Information S1. Interestingly, the evidence reported in Table S1.10 shows that the trend of the results obtained in other impact categories is the same as the trend followed by GWP results. The only exception to this can be noticed for the acidification potential in bespoke chandeliers: here, the highest and intermediate values are swapped between Chandeliers 1 and 3. This is due to a higher impact in this category for stages A1 and A3 for Chandelier 3, and it is most likely related to this chandelier requiring the greatest amount of materials among the three bespoke products, therefore generating more burdens during manufacturing.

Future research on these topics should focus on improvement to the TM65 methodology, especially applied to the lighting industry, in order to limit the overestimation of the embodied carbon of products. Strategies that could help achieve this, without compromising the simplicity of the approach, include: (i) the addition of more embodied carbon coefficients for materials typically used in lighting manufacturing; (ii) clarity regarding the inclusion/exclusion of power supplies and light sources in a consistent way, as these components have the highest embodied carbon coefficients and provide major contributions to the total impact of a product; and (iii) the possibility of accounting for manufacturing operations in a more accurate way rather than based on the product’s weight—that is, factoring in a realistic supply chain of materials and components. To identify further critical aspects, the method presented in this study could be applied to look at other lighting manufacturers using different techniques and/or supply chains or focusing on a range of different lighting products, with the aim of better understanding how these factors and differences can be best modeled to represent them.

## CONCLUSIONS

The results of the cradle-to-grave embodied carbon analysis showed an overestimate of the values obtained with TM65 compared to the LCA methodology, in accordance with the results reported in TM65 (Hamot & Bagenal George, 2021). The discrepancies in the resultant values were larger for bespoke projects, with an average of 96% discrepancy between the three bespoke chandeliers. The average difference between the two methodologies for standard products was 68%. The life cycle stages playing a determining role in result discrepancies for bespoke projects are the material extraction (A1), manufacturing (A3), and EoL stages (C2–C4). The manufacturing (A3) and EoL (C2–C4) stages contribute to a lesser extent to the difference between the calculations for standard products, as opposed to bespoke projects, whilst material extraction (A1) and repair (B3) stages contributed substantially to increase the gap between results. These observations led to the conclusion that the way stages A1, A3, B3, and C2–C4 are accounted for in TM65, that is, considering them as dependent on the weight of the products, causes the method to overestimate the embodied carbon values.

For a company, the advantages of using TM65 for embodied carbon calculations include the adaptability of the method to different categories of products, the simplicity of the calculations, its time efficiency, and the presence of an active community working to improve and update the methodology. However, the simplicity and high adaptability of TM65 can sometimes result in low accuracy, in particular for processes that are exclusive to a certain family of products (e.g., lighting). On the other hand, the LCA methodology requires a higher level of skills and knowledge to be applied, with the use of specific software and databases and it is a more time-consuming methodology. Furthermore, the risks associated with an LCA are mostly related to the subjectivity of the analysis. For this reason, PCRs and product specific rules (PSRs) must be followed when carrying out LCAs to produce EPDs. Nevertheless, due to the possibility to include highly specific information, the LCA methodology can provide more accurate results. Bearing in mind that the scope of TM65 is primarily to bridge the gap between industry and sustainability assessment, this study shows that the trend followed by the embodied carbon results is the same between the LCA and TM65 methods. This confirms the validity of TM65 as a comparative methodology to identify low/high carbon products.

In conclusion, both methodologies show a trade-off between accuracy and simplicity. The authors believe that TM65, especially the mid-level calculation, is a valid embodied carbon estimate method while the industry starts producing EPDs in a consistent way, as the creators of the methodology suggest. However, given the results of this study, improvements to the methodology could be made, by incorporating suggestions from the industry, as more manufacturers start applying it consistently to their production, and by updating the methodology based on new data availability and research findings.

## Supplementary Information

**Supporting Information S1**: This supporting information provides additional data and information on the Life Cycle Inventory and the datasets used to carry out the analysis (Section S.1), the energy consumed during the in-use stage included in the whole-life LCA analysis (Section S.2), the results of the whole-life LCA with modified scope and trend followed by results (Section S.3) and the results of the whole-life LCA including additional flows for full compliance with EN 15804 (Section S.4).

**Supporting Information S2**: This supporting information provides life cycle inventory data common to TM65 and LCA (Tables S2.1 and S2.2), manufacturing flows used in LCA (Tables S2.3 and S2.4), life cycle impact assessment results obtained through the application of the CML baseline method (Table S2.5 and S2.6), additional LCI data to achieve full compliance with EN 15804 requirements (Table S2.7 and S2.8) and relative LCIA results (Table S2.9 and S2.10).

## Data Availability

The data that supports the findings of this study are available in the supporting information of this article.
