# Supplementary material for: Embodied carbon quantification of luminaires using life cycle assessment and CIBSE TM65 methodologies: A comparison case study
Source: J Ind Ecol. 2023 Oct 13;28(1):59–73. doi: 10.1111/jiec.13449 (PMC13128737; doi:10.1111/jiec.13449)
Supplement: Supplementary file 1 — Supporting Information S1: This supporting information provides additional data and information on the Life Cycle Inventory and the datasets used to carry out the analysis (Section S.1), the energy consumed during the in-use stage included in the whole-life LCA analysis (Section S.2), the results of the whole-life LCA with modified scope and trend followed by results (Section S.3) and the results of the whole-life LCA including additional flows for full compliance with EN 15804 (Section S.4). [file 44498_2024_2801005_MOESM1_ESM.docx]

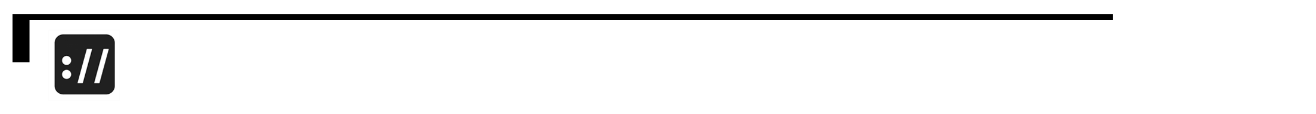


SUPPORTING INFORMATION FOR:

Mazzei, I., Saint, R., Kay, A. & Pomponi, F. (2023) Embodied Carbon Quantification of Luminaires using Life Cycle Assessment and CIBSE TM65 Methodologies: A Comparison Case Study. *Journal of Industrial Ecology.*


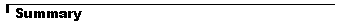


This supporting information provides additional data and information on the Life Cycle Inventory and the datasets used to carry out the analysis (Section S.1), the energy consumed during the in-use stage included in the whole-life LCA analysis (Section S.2), the results of the whole-life LCA with modified scope and trend followed by results (Section S.3) and the results of the whole-life LCA including additional flows for full compliance with EN 15804 (Section S.4).


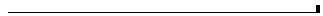


# **Life Cycle Inventory**

| 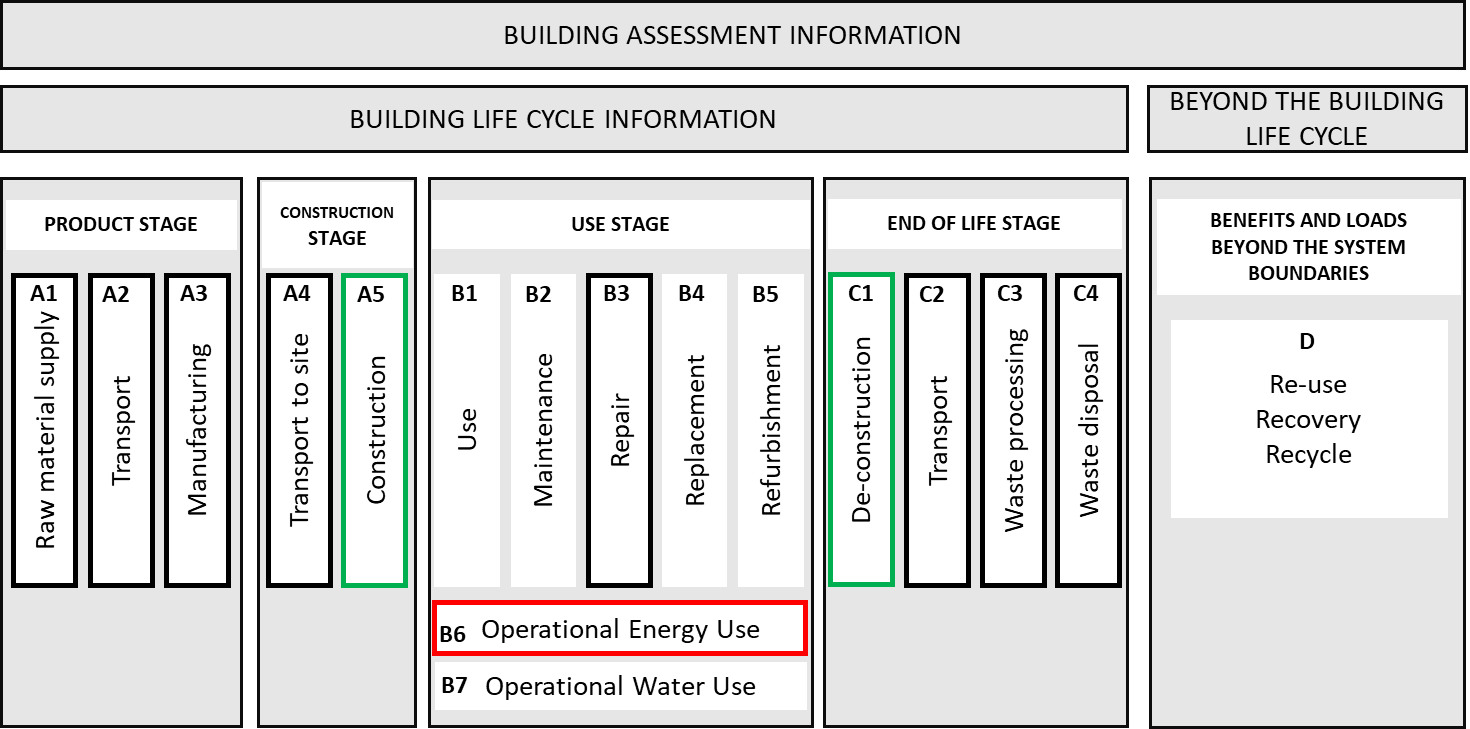 |
| --- |
| **Figure S1.1**: Life cycle stages of a product (CEN, 2019); black thick lines indicate life cycle stages included in the analysis comparing TM65 and LCA with modified scope, red thick lines indicate additional life cycle stages included for whole-life LCA analysis and green thick lines indicate additional life cycle stages further included to show results compliant with EN 15804 standard. |

**Table S1.1**: Values of X in the definition of declared unit (a luminaire providing a luminous flux of X lumens during a reference life of 25 years), for each product.

|  | Standard | | |  | Bespoke | | | |
| --- | --- | --- | --- | --- | --- | --- | --- | --- |
|  | Fitting 1 | Fitting 2 | Fitting 3 | | Chandelier 1 | Chandelier 2 | Chandelier 3 |  |
| Value of X (lm) | 100 | 873 | 1421 | | 97980 | 120509 | 160000 |  |

**Table S1.2**: Embodied carbon coefficients of materials used in the TM65 assessment.

| Material | Embodied Carbon Coefficient (kg CO_2_ eq./kg) | Source |
| --- | --- | --- |
| Recycled Aluminium | 1.69 | (Hammond & Jones, 2011) |
| Nylon 6-6 | 6.54 |  |
| ABS | 3.76 | (CIBSE, 2021), (Hammond & Jones, 2019), (CCaLC, *nd*) |
| Aluminium | 13.10 |  |
| Brass | 4.80 |  |
| Copper | 3.81 |  |
| Glass | 1.44 |  |
| Insulation | 1.86 |  |
| Plastics | 3.31 |  |
| Polycarbonate | 7.62 |  |
| Rubber/Silicone | 2.85 |  |
| Stainless Steel | 4.40 |  |
| Steel | 2.97 |  |
| Electronic components | 49.0 |  |
| Printed circuit board | 154.0 |  |

**Table S1.3**: Embodied carbon coefficients of electricity and gas used in the TM65 assessment.

| Quantity | Embodied Carbon Coefficient  kg CO_2_ eq/kWh | Source |
| --- | --- | --- |
| Electricity^a^ | 0.29 | *(UK Government, 2020)* |
| Gas^b^ | 0.23 |  |

^a^ UK Electricity generation, transmission and distribution including well-to-tank emissions.
^b^ Emissions for net calorific value including well-to-tank emissions.

**Table S1.4**: Electricity and gas usage corresponding to each product estimated from the total energy usage and production of the factory and used in TM65 calculations.

| Product | Electricity usage (kWh) | Gas usage (kWh) |
| --- | --- | --- |
| Fitting 1 | 0.34 | 0.49 |
| Fitting 2 | 1.73 | 2.49 |
| Fitting 3 | 4.73 | 6.80 |
|  |  |  |
| Chandelier 1 | 1214.75 | 1744.63 |
| Chandelier 2 | 1726.05 | 2478.97 |
| Chandelier 3 | 2657.94 | 3817.36 |

**Table S1.5**: Transport data used for the LCA analysis in relation to stage A4.

| Product | Ecoinvent data | Distance (km) | Quantity (tkm) |
| --- | --- | --- | --- |
| Fitting 1 | Transport, freight, lorry 7.5-16 metric ton, EURO5 {RER}\| Cut-off, U | 650 | 0.06 |
| Fitting 2 |  |  | 0.28 |
| Fitting 3 |  |  | 0.78 |
|  |  |  |  |
| Chandelier 1 | Transport, freight, aircraft, long haul {GLO}\| Cut-off, U | 5530 | 1694.19 |
| Chandelier 2 | Transport, freight, lorry 7.5-16 metric ton, EURO5 {RER}\| Cut-off, U | 1500 | 653.32 |
| Chandelier 3 |  | 530 | 355.28 |

**Table S1.6**: Waste stream mixes for relevant materials.

| Material | Recycled (%) | Incinerated (%) | Landfill (%) | Source |
| --- | --- | --- | --- | --- |
| Aluminium | 98.0 | 2.0 | - | (*Recolight*, n.d.) |
| Ferrous metals | 98.0 | 2.0 | - |  |
| Plastics | 57.1 | 42.9 | - |  |
| LED | 90.0 | 10.0 | - |  |
| Glass | 90.0 | 10.0 | - |  |
| Electronics | 40.0 | - | 60.0 | (*European Parliament*, 2020) |
| Neodymium | 100.0 | - | - | (Jin et al., 2018) |

**Table S1.7**: Processes used for the end-of-life stage in the LCA analysis.

| Ecoinvent Process | Unit |
| --- | --- |
| aluminium scrap, post-consumer, prepared for melting {CH}\| treatment of metal scrap, mixed, for recycling, unsorted, sorting \| Cut-off, U | kg |
| Electronics scrap from control units {RER}\| treatment of \| Cut-off, U |  |
| EoL, Professional Lighting Equip. \| PCB Support, Substitution benefits not included |  |
| Inert waste, for final disposal {CH}\| treatment of inert waste, inert material landfill \| Cut-off, U |  |
| Waste plastic, mixture {CH}\| treatment of, municipal incineration \| Cut-off, U |  |
| Municipal solid waste {GLO}\| treatment of, incineration \| Cut-off, U |  |
| Mixed plastics (waste treatment) {GLO}\| recycling of mixed plastics \| Cut-off, U |  |

**Table S1.8**: Impact categories considered in the LCA analysis.

| Impact Category | Unit | Abbreviations |
| --- | --- | --- |
| Global Warming Potential | Kg CO_2_ eq. | GWP |
| Acidification Potential | Kg SO_2_ eq. | AP |
| Resource Depletion Potential – Abiotic resources | Kg Sb eq. | RDP – A |
| Resource Depletion Potential – Fossil Fuels | MJ | RDP – FF |
| Ecotoxicity Potential – Fresh water | Kg 1,4-DB eq. | EP – F |
| Ecotoxicity Potential – Marine | Kg 1,4-DB eq. | EP – M |
| Ecotoxicity Potential – Terrestrial | Kg 1,4-DB eq. | EP – T |
| Ecotoxicity Potential – Human | Kg 1,4-DB eq. | EP – H |
| Ozone Depletion Potential | Kg CFC-11 eq. | ODP |
| Photochemical Oxidation Potential | Kg C_2_H_4_ eq. | POP |
| Eutrophication Potential | Kg PO_4_^3-^ eq. | EuP |

# **Energy in Use (B6)**

The energy consumed during the use stage (B6) was modelled in the LCA considering the average electricity production sources for the Countries involved in the study where the final user was based (England, France or United States). Details of the electricity mixes used in the calculations are reported in **Table S1.9**. No decarbonisation of the grid in those countries has been factored in for the 25 years of operation, thus overestimating (and potentially to a significant extent, since electricity is easier to decarbonise than heating) the B6 impact.

**Table S1.9** Electricity mixes used for manufacturing and in-use stages. Values were rounded up to zero decimal places.

| Life Cycle Stage | Country | Electricity Mix | Source |
| --- | --- | --- | --- |
| Manufacturing (A3) | Scotland | *100% Renewable* ^a^  62% Onshore Wind; 11% Offshore Wind; 19% Hydro;  8% Other (e.g. Photovoltaic) | (The Scottish energy statistics hub, 2021) |
| Operational Energy Use  (B6) | England *^b^* | 36% Gas; 28% Wind; 16% Nuclear; 13% Photovoltaic; 3% Oil; 2% Hydro; 2% Coal | (BEIS, 2021) |
|  | France | 66% Nuclear; 13% Hydro; 8% Wind; 7% Gas; 3% Photovoltaic; 1% Biogas; 1% Oil; 1% Coal | (*IEA, 2020)* |
|  | USA | 38% Gas; 22% Coal; 19% Nuclear; 9% Wind; 6% Hydro;  3% Photovoltaic; 1% Oil; 2% Others | (*EIA, 2021)* |

^a^ Based on electricity provider’s information.

^b^ England was used as customer site for catalogue products, based on company market data provided by Stoane Lighting Ltd.

# **LCA – Whole-life Environmental Impact Assessment**

The results of the LCIA on the data, obtained using the CML baseline method, are shown in **Figure S1.2**, and in **Table S2.5** and **Table S2.6** in Supporting Information S2. The results are reported at the product level, for each life cycle stage of the products.

| 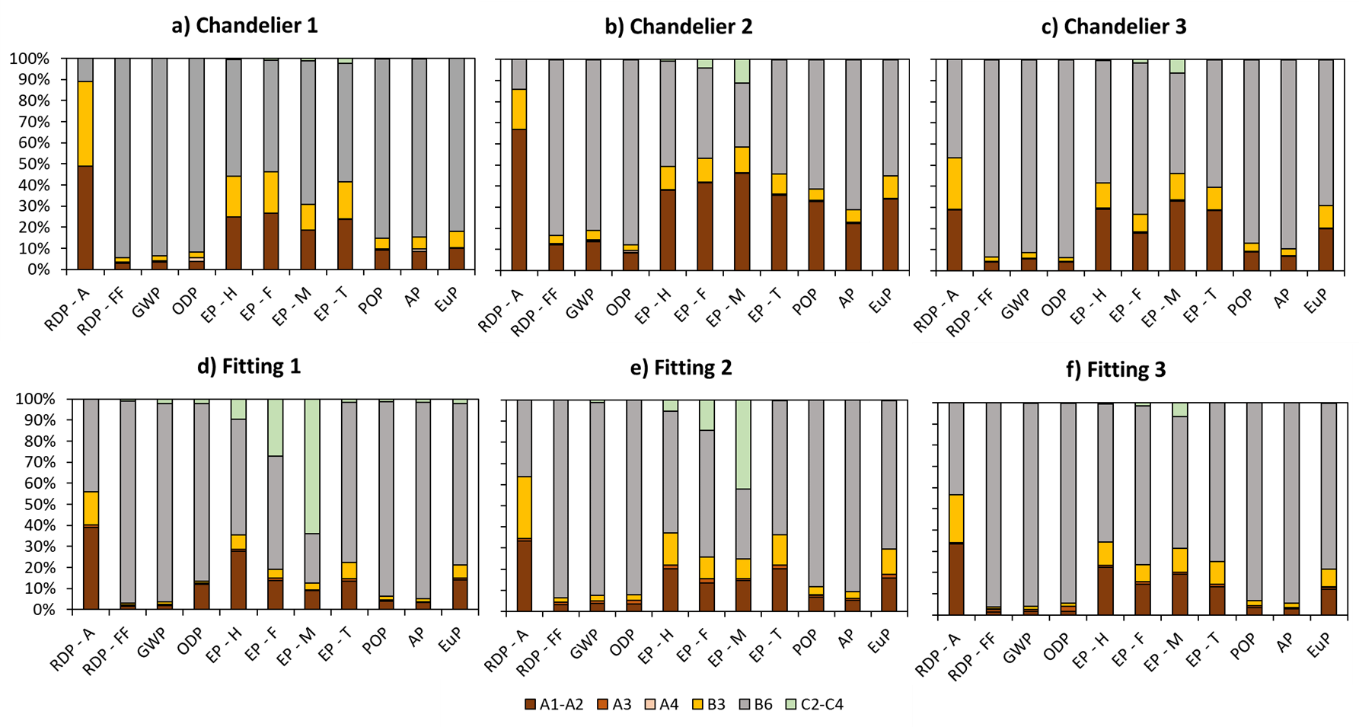 |
| --- |
| **Figure S1.2**: Life cycle assessment results obtained with the CML-IA baseline method applied to the LCI. |

The trend followed by the results in all impact categories is shown in **Table S1.10**. The orange and green cells represent the lowest and highest values for each impact category in each set of products, respectively, whilst the yellow cells represent the average between the lowest and highest values. The table shows that the trend observed for the GWP is the same in the other impact categories as well, with the only exception of the acidification potential in bespoke chandeliers which shows an inverted trend.

**Table S1.10:** Impact results calculated with the CML baseline method in each impact category, for each product. Colour coding indicates highest (green), lowest (orange) and average (yellow) values.

|  | Fitting 1 | Fitting 2 | Fitting 3 |  | Chandelier 1 | Chandelier 2 | Chandelier 3 |
| --- | --- | --- | --- | --- | --- | --- | --- |
| RDP - A | 8.49E-04 | 3.15E-03 | 7.36E-03 |  | 2.10E+00 | 9.80E-01 | 1.38E+00 |
| RDP - FF | 5.91E+02 | 1.86E+03 | 5.08E+03 |  | 1.56E+06 | 2.85E+05 | 1.05E+06 |
| GWP | 4.00E+01 | 1.27E+02 | 3.38E+02 |  | 1.15E+05 | 2.19E+04 | 7.12E+04 |
| ODP | 3.84E-06 | 1.08E-05 | 2.96E-05 |  | 7.70E-03 | 2.36E-03 | 5.99E-03 |
| EP - H | 2.99E+01 | 8.77E+01 | 2.18E+02 |  | 6.50E+04 | 2.76E+04 | 4.88E+04 |
| EP - F | 4.55E+01 | 1.25E+02 | 2.82E+02 |  | 5.96E+04 | 2.47E+04 | 5.91E+04 |
| EP - M | 1.07E+05 | 2.32E+05 | 3.45E+05 |  | 1.69E+08 | 4.95E+07 | 9.06E+07 |
| EP - T | 3.14E-02 | 1.15E-01 | 2.73E-01 |  | 8.63E+01 | 4.08E+01 | 6.80E+01 |
| POP | 7.33E-03 | 2.36E-02 | 6.25E-02 |  | 1.60E+01 | 4.87E+00 | 1.34E+01 |
| AP | 1.65E-01 | 5.22E-01 | 1.40E+00 |  | 2.61E+02 | 8.43E+01 | 2.97E+02 |
| EuP | 3.16E-02 | 1.06E-01 | 2.65E-01 |  | 1.53E+02 | 3.16E+01 | 6.02E+01 |

# **LCA – Whole-life Environmental Impact Assessment with EN 15804 additions**

Data to represent life cycle stages A5 (installation) and C1 (deconstruction) and inclusion of manufacturing waste and packaging were added to the LCI, according to the rules in standard EN 15804, and are reported in **Table S2.7** and **Table S2.8** in Supporting Information S2. The results obtained using the CML baseline method applied to the data are shown in **Figure S1.3**, and in **Table S2.9** and **Table S2.10** in Supporting Information S2. The results are reported at the product level, for each life cycle stage of the products.

| 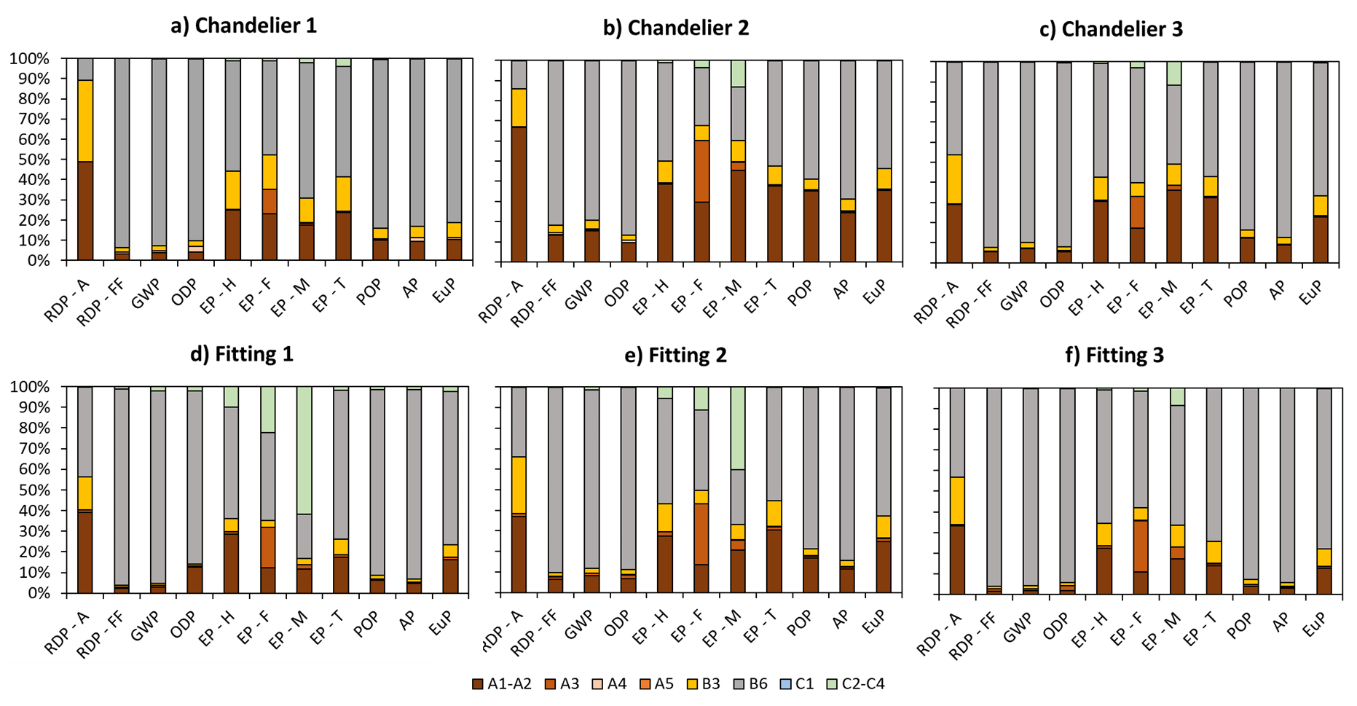 |
| --- |
| **Figure S1.3:** Life cycle assessment results obtained with the CML-IA baseline method applied to the LCI, with additional data according to EN 15804. |

The GWP values of catalogue and bespoke products obtained with LCA with modified scope and compliant with EN 15804 are reported in **Table S1.11**, showing an average difference of 2.1% and maximum 4.9% for Fitting 2.

**Table S1.11**: Results of the LCA with modified scope to align to TM65 in comparison to a more complete LCA where additional flows and life cycle stages were added to achieve compliance with EN 15804.

|  | GWP (100y) (kg CO_2_ eq.) | | Difference  (kg CO_2_ eq.) | Difference  (%) |
| --- | --- | --- | --- | --- |
|  | Modified scope | EN15804-compliant |  |  |
| Chandelier 1 | 114915.9 | 116027.4 | 1111.4 | 1.0 |
| Chandelier 2 | 21884.0 | 22373.8 | 489.8 | 2.2 |
| Chandelier 3 | 71181.9 | 72570.5 | 1388.6 | 1.9 |
|  | | | | |
| Fitting 1 | 40.0 | 40.6 | 0.5 | 1.3 |
| Fitting 2 | 126.9 | 133.3 | 6.4 | 4.9 |
| Fitting 3 | 338.4 | 339.9 | 1.5 | 0.4 |
|  |  |  | Average | 2.1 |

# REFERENCES

CEN. (2019). *EN 15804:2012 + A2:2019*. <https://bsol-bsigroup-com.napier.idm.oclc.org/PdfViewer/Viewer?pid=000000000030442810>

CIBSE. (2021). *CIBSE TM65 - Embodied carbon in building services: a calculation methodology*.

*Carbon Calculations over the Life Cycle of Industrial Activities*. (n.d.). Retrieved June 8, 2023, from <http://www.ccalc.org.uk/index.php>

Hammond, G., & Jones, C. (2011). *The Inventory of Carbon and Energy (ICE)*.

Hammond, G., & Jones, C. (2019). *Embodied Carbon Footprint Database - ICE*. <https://circularecology.com/embodied-carbon-footprint-database.html>

*UK Government*. (2020). <https://www.gov.uk/government/publications/greenhouse-gas-reporting-conversion-factors-2020>

*Recolight*. (n.d.). Retrieved October 3, 2022, from <https://www.recolight.co.uk/weee-info/how-we-recycle/>

*European Parliament*. (2020). <https://www.europarl.europa.eu/news/en/headlines/society/20201208STO93325/e-waste-in-the-eu-facts-and-figures-infographic>

Jin, H., Afiuny, P., Dove, S., Furlan, G., Zakotnik, M., Yih, Y., & Sutherland, J. W. (2018). Life Cycle Assessment of Neodymium-Iron-Boron Magnet-to-Magnet Recycling for Electric Vehicle Motors. *Environmental Science and Technology*, *52*(6), 3796–3802. <https://doi.org/10.1021/ACS.EST.7B05442/SUPPL_FILE/ES7B05442_SI_003.XLSX>

The Scottish energy statistics hub. (2021). *Energy Statistics for Scotland Q4 2020 Figures Overall* . <https://www.gov.scot/binaries/content/documents/govscot/publications/statistics/2018/10/quarterly-energy-statistics-bulletins/documents/energy-statistics-summary---march-2021/energy-statistics-summary---march-2021/govscot:document/Scotland+Energy+Statistics+Q4+2020.pdf>

BEIS. (2021). *UK Energy in Brief 2021*. [www.gov.uk/government/statistics/uk-energy-in-brief-2021](http://www.gov.uk/government/statistics/uk-energy-in-brief-2021)

IEA. (2020). *France - Countries & Regions - IEA*. <https://www.iea.org/countries/france>

EIA. (2021). *U.S. Electricity Generation by Energy Source 2021 - U.S. Energy Information Administration (EIA)*. <https://www.eia.gov/tools/faqs/faq.php?id=427&t=3>
